# Patient-reported outcomes, provider-reported outcomes, and physiologic parameters after gender-affirming hormone treatment in Canada: a systematic review

**DOI:** 10.1007/s42000-024-00626-y

**Published:** 2025-01-08

**Authors:** Liam Jackman, Cynthia Chan, Micon Garvilles, Rakhshan Kamran

**Affiliations:** 1 Institute of Health Policy, Management and Evaluation, University of Toronto, Toronto, ON Canada; 2 Department of Family and Community Medicine, University of Toronto, Toronto, ON Canada; 3 Nuffield Department of Orthopaedics, Rheumatology and Musculoskeletal Sciences, University of Oxford, Oxford, OX37LD UK

**Keywords:** Patient-reported outcome measures, PROMs, Provider-reported outcomes, Physiologic parameters, Gender-affirming care, Hormone therapy, Health services for transgender persons, Health services research, Systematic review

## Abstract

**Purpose:**

Canada has experienced a ten-fold increase in referrals for gender-affirming care. Clinical guidelines emphasize the importance of a comprehensive and systematic approach to outcome measurement for gender-affirming hormonal care. However, research is lacking on the investigation of outcomes of Canadian gender-affirming hormonal treatments.

**Methods:**

In total, five databases were searched, as follows: MEDLINE, Embase, PsycINFO, Scopus, and CINAHL on December 26, 2023. To meet inclusion criteria, each study needed to be an original article including patients identifying as transgender or gender diverse (TGD) who were receiving gender-affirming hormonal care in Canada. The Critical Appraisal Skills Programme (CASP) and Joanna Briggs Institute (JBI) tools were used to assess the methodological quality of the study. Descriptive frequencies were calculated for demographic information and a narrative synthesis was conducted to summarize outcomes organized for different treatments.

**Results:**

A total of 3315 articles were identified, with 34 articles being included, representing 3990 patients. Physiologic parameters were reported in 62% of the studies and patient-reported outcomes (PROs) in 50%, although only 32% utilized standardized patient-reported outcome measures (PROMs). In studies reporting quantitative results, testosterone treatments showed 80% effectiveness in achieving desired physical changes, while several studies demonstrated that estrogen and antiandrogen treatments improved mental health in 85% of patients. The narrative synthesis of the results reveals positive outcomes and limited adverse effects of gender-affirming hormone therapy; however, it also underscores the need for standardized, consistent outcome measurement tools, particularly PROMs.

**Conclusion:**

The present systematic review highlights the need for standardized outcome reporting in gender-affirming hormone therapy, underscoring the need for measurement of the patient’s perspective through PROMs. Resolving these issues can improve evidence-based practices and support high-quality, patient-centered gender-affirming hormone care.

**Supplementary Information:**

The online version contains supplementary material available at 10.1007/s42000-024-00626-y.

## Introduction

Gender-affirming care refers to a range of psychosocial, hormonal, and surgical treatments aimed at alleviating gender dysphoria, that is, the discomfort or distress experienced due to incongruence between one’s gender and one’s sex assigned at birth [1]. Over the past decade, Canada has seen a ten-fold increase in referrals for gender-affirming care services, this paralleling a global increase in demand for these services [2–4] However, while clinical guidelines and clinical standards consistently call for a comprehensive, evidence-based approach to outcome measurement across all aspects of gender-affirming care [1], a previous systematic review has revealed a scarcity of Canadian research specifically on outcome measurement after gender-affirming hormonal care [5].

The scope of health outcome measurement spans performance indicators (e.g., wait time), physiologic parameters (e.g., hormone concentrations), provider-reported outcomes (e.g., surgical complications), and patient-reported outcomes (e.g., quality of life) [6]. Ideally, patient-reported outcomes should be measured using patient-reported outcome measures (PROMs), which are standardized self-report tools [7, 8]. PROMs data can be used to guide treatment selection and measure treatment effectiveness [9] and may also be used in comparative treatment- and cost-effectiveness research [8].

Unfortunately, despite calls for a comprehensive, evidence-based approach to outcome measurement, there continues to be an overemphasis on physiologic parameters, performance indicators, and provider-reported outcomes and an underemphasis on patient-reported outcomes (particularly on those captured using PROMs). This may result in discrepancies between the care provided by a healthcare provider and the actual preferences of a patient, thus diminishing patient-centered care [5, 10]. The aim of this systematic review is to comprehensively analyze outcomes measured and outcomes reported after gender-affirming hormonal care in Canada. Such information is needed to provide informed recommendations for the improvement of outcome measurement practices.

## Methods

### Ethics

This study was reviewed by the University of Oxford and deemed exempt from University sponsorship or ethical review as no human data was collected; it is a review of published literature.

### Patient and public involvement

The current study was conducted in partnership with seven transgender and gender diverse (TGD) individuals. These individuals confirmed the importance of the research, confirmed the comprehensiveness of the search, and provided feedback throughout to augment the applicability of the research findings.

### Reporting

The present systematic review is compliant with the Preferred Reporting Items for Systematic Reviews and Meta-Analysis (PRISMA) guidelines [11]. The protocol for this review was also prospectively registered (PROSPERO CRD42023462839).

### Search strategy

The search strategy (presented in the Supplementary File) was developed with assistance from an information scientist outside of the author team. The search was a part of a larger study evaluating outcome measurement practices for gender-affirming care in Canada which included psychosocial, medical, and surgical care. The current paper focuses on outcome measurement after gender-affirming medical care (i.e., hormonal care).

### Information sources and eligibility criteria

In total, five electronic databases were searched, as follows: MEDLINE, Embase, PsycINFO, Scopus, and CINAHL. The search was conducted on December 26, 2023, with no language restrictions. Articles were included 1) if they concerned an original article, 2) when the patients identified as TGD, and 3) when the patients were accessing/receiving gender-affirming care in Canada. Articles were excluded 1) if they concerned a non-original article, 2) when the patients were not identified as TGD, and 3) when the patients were not accessing gender-affirming care in Canada.

### Selection, data collection, and data items

Titles, abstracts, and full-texts were screened independently and in duplicate on Covidence (RK, LJ, CC, and MG), with conflicts resolved by a third reviewer (LJ). Data extraction conducted independently and in duplicate (RK, LJ, CC, and MG), with conflicts resolved by a third reviewer (LJ). Data items extracted included the following: general information (DOI, title, year, first author, first author affiliation, article type, study aim, study design, and study funding), population information (recruitment, inclusion criteria, exclusion criteria, number of participants, gender identity of participants, race/ethnicity of participants, age, country, province, city, and gender-affirming care provided), and study outcomes (health outcome measure, barriers and enablers to health outcome measurement, barriers and enablers to accessing gender-affirming care, researcher reflexivity, patient and public involvement, and data security).

### Study risk of bias assessment

The Critical Appraisal Skills Programme (CASP) tool [12] was used to assess study quality, except for case series/studies where the Joanna Briggs Institute (JBI) Critical Appraisal Tool [13] was used (CASP checklist not available for these). This assessment of the study was conducted independently and in duplicate (RK, LJ, CC, and MG), with conflicts resolved by a third reviewer (LJ).

### Synthesis methods

Descriptive frequencies were used to analyze demographic data. Narrative synthesis was used to synthesize outcome data. Narrative synthesis was conducted in accordance with the SWiM (Synthesis Without Meta-Analysis) guidelines [14].

## Results

### Study selection

**Fig. 1:**
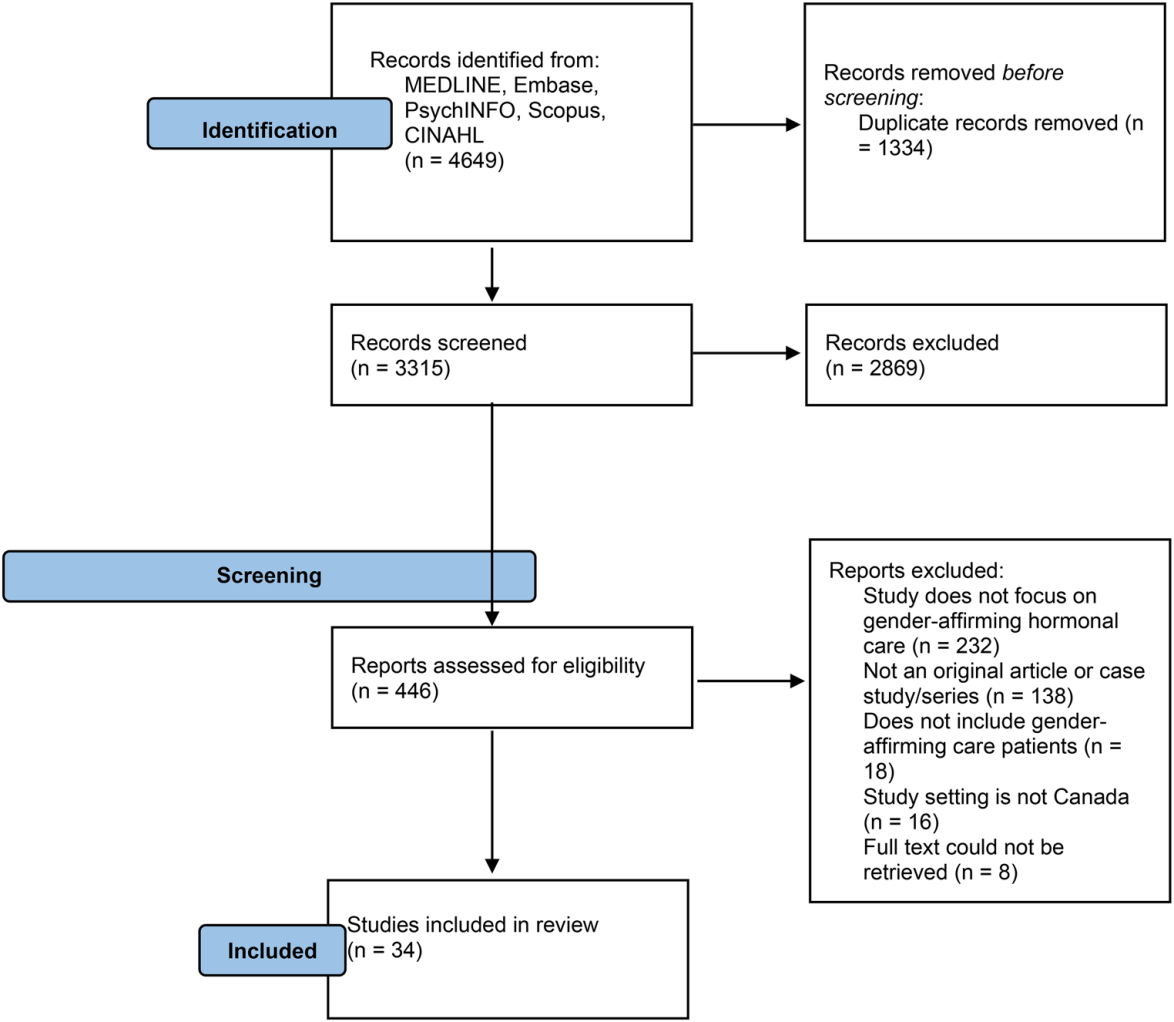
PRISMA diagram of study selection

In total, 3315 articles were identified from the search, 34 of which were deemed eligible for this article [15–48]. Figure 1 displays the PRISMA diagram for the current study. According to the PRISMA guidelines, reasons for title/abstract exclusions are not required. However, reasons for full-text exclusion are required [11]. We followed the established PRISMA guidelines and reasons for full-text exclusion are presented in Fig. 1.

### Patient characteristics

In total, 3990 patients receiving gender-affirming hormones are represented in this systematic review. The 34 included articles reported race, ethnicity, and gender inconsistently. In total, eight studies reported race and/or ethnicity. However, among these, a significant amount of race and/or ethnicity data was missing (42%). Table 1 provides data on race/ethnicity and gender of included participants reported employing the terms used in the included articles. Among studies reporting mean age, the mean age ranged from 13.7 to 54.8. Among studies reporting minimum and maximum ages, the age ranged from 4.7 to 70.

**Table 1:** Gender and race/ethnicity of included participants

Race/ethnicity (reported in eight studies)		
**Race/ethnicity**	**Frequency**	%
Aboriginal	30	1.3
Asian	71	3.1
Black	41	1.8
Black, African, Caribbean	5	0.2
Caucasian, Irish, Canadian, Quebecois	24	1.0
East Asian	18	0.8
First Nation	38	1.7
Indigenous	79	3.4
Jewish	4	0.2
Latinx	42	1.8
Missing	966	42.0
Non-First Nation	123	5.3
Non-White or Indigenous	4	0.2
Non-Indigenous visible minority	10	0.4
Non-Indigenous White	128	5.6
NR	7	0.3
Other	12	0.5
Other or mixed	78	3.4
South Asian	41	1.8
Three or more ethnicities	11	0.5
White	570	24.8
**Gender**		
**Gender**	**Frequency**	**%**
Assigned female at birth, masculine gender identity	23	0.6
Assigned male at birth, feminine gender identity	10	0.3
Female	55	1.4
Female or primarily a girl	64	1.6
Female to male gender reassignment	1	0.0
Female to male transgender	4	0.1
Female to male transsexual	32	0.8
Genetic male transsexual	13	0.3
Male	54	1.4
Male or primarily a boy	251	6.3
Male to female transgender	11	0.3
Male to female transsexual	36	0.9
Natal female	122	3.1
Natal females female to male	45	1.1
Natal male	52	1.3
Natal males “unsettled in gender identity”	2	0.1
Natal males male to female	37	0.9
Nonbinary	377	9.4
Other	3	0.1
Trans boy/man	356	8.9
Trans female/trans feminine	35	0.9
Trans girl/woman	139	3.5
Trans male/trans masculine	46	1.2
Trans woman	1768	44.3
Transfeminine	14	0.4
Transfeminine nonbinary or similar	3	0.1
Transfeminine woman	53	1.3
Transgender	15	0.4
Transgender female	52	1.3
Transgender male	135	3.4
Transmasculine	78	2.0
Transmasculine	22	0.6
Transsexual man	50	1.3
Transsexual woman	32	0.8

### Study information

In general, the number of publications increased each year (from 1986 to 2023), with the highest proportion of articles published in 2021 (18%). Most articles were cohort studies (65%) and had received funding (50%). Most studies were conducted in Ontario (41%), with Toronto being the most frequently studied city (29%). Study information is presented in Table 2.

**Table 2:** Study information for included articles

Year of study publication		
**Year**	**Frequency**	%
1986	1	2.9
1989	1	2.9
1995	1	2.9
2000	2	5.9
2007	1	2.9
2011	1	2.9
2014	1	2.9
2015	1	2.9
2016	2	5.9
2017	2	5.9
2018	4	11.8
2019	3	8.8
2020	1	2.9
2021	6	17.6
2022	2	5.9
2023	5	14.7
**Type of study**		
**Type of study**	**Frequency**	**%**
Case series/case report	7	20.6
Cohort study	22	64.7
Cross-sectional	3	8.8
Qualitative	1	2.9
Mixed methods	1	2.9
**Funding information**		
**Funding**	**Frequency**	**%**
Yes	17	50.0
No	9	26.5
NR	8	23.5
**Province of study**		
**Province**	**Frequency**	**%**
Alberta	2	5.9
Alberta, British Columbia, Manitoba, Nova Scotia, Ontario, Quebec, Saskatchewan	1	2.9
British Columbia	6	17.6
Manitoba	1	2.9
Nova Scotia, Quebec, Ontario, Manitoba, Alberta, British Columbia	1	2.9
Ontario	14	41.2
Ontario, British Columbia, Quebec	1	2.9
Quebec	4	11.8
Quebec, Ontario	1	2.9
Unspecified/NR	3	8.8
**City of study**		
**City**	**N**	**%**
Edmonton	2	5.9
Halifax, Montreal, Ottawa, Toronto, Hamilton, London, Winnipeg, Calgary, Edmonton, Vancouver	1	2.9
Kingston	1	2.9
Montreal	4	11.8
Montreal, Toronto	1	2.9
Ottawa	2	5.9
Toronto	10	29.4
Toronto, Vancouver, Montreal	1	2.9
Unspecified/NR	6	17.6
Vancouver	5	14.7
Winnipeg, rural Manitoba, First Nations reserve, other	1	2.9

### Outcomes measured

Physiologic parameters were reported in 62% of studies. Patient-reported outcomes were reported in 50% of the studies; however, among these studies, PROMs were used in only 32% of them. The actual PROMs administered across these studies were inconsistent. Table 3 provides an overview of outcomes measured and PROMs used across included articles. Table 4 provides an overview of clinical and endocrinological parameters of gender-affirming hormone therapy from the included articles. Details of these parameters and outcomes are discussed below.

**Table 3:** Overview of outcomes measured and PROMs used across articles

Outcomes reported among articles		
Outcomes reported	*N*	%
Physiologic parameter	14	41.2
Patient-reported outcome	11	32.4
Physiologic parameter, patient-reported outcome	3	8.8
Physiologic parameter, provider-reported outcome	3	8.8
Physiologic parameter, provider-reported outcome, patient-reported outcome	1	2.9
Provider- reported outcome, patient-reported outcome	2	5.9
**PROM use among studies**		
**Was a PROM used?**	**N**	**%**
Yes	11	32.4
No	23	67.6
**PROM used**		
**PROM**	**Frequency**	%
HIV Stigma Scale [49]	1	4
Adult Self-Report Questionnaire [50]	1	4
Canadian Community Health Survey [51]	2	8
Canadian Trans Youth Health Survey [52]	1	4
Child Behaviour Checklist (CBCL) (parent-report questionnaire) [53]	1	4
Erotic Response and Orientation Scale (EROS) [54]	1	4
Gender Identity Questionnaire for Adolescents (GIQ) [55]	2	8
Gender Identity/Gender Dysphoria Questionnaire for Adolescents and Adults (GIDYQ-AA) [56]	1	4
Migraine Disability Assessment Scores (MIDAS) [57]	1	4
Physical Health Component (PCS) Summary Score of the Short Form (SF) Health Survey [58]	1	4
Stressors on Families of Trans Youth Checklist [59>]	1	4
Telehealth Usability Questionnaire (TUQ) [60]	1	4
Modified Depression Scale [61]	2	8
Overall Anxiety Severity and Impairment Score (OASIS) [62)]	2	8
Kessler-6 Scale for Psychological Distress [63]	1	4
Piers-Harris Children Self Concept Scale [64]	1	4
Trans Youth CAN! Gender Distress Scale [32]	1	4
Utrecht Gender Dysphoria Scale [65]	1	4
Trans Voice Questionnaire [66]	2	8
Youth Self-Report (YSR) [67]	1	4

**Table 4:** Clinical and endocrinological parameters of gender-affirming hormone therapy from included articles

Citation	Study aim	Number of patients and gender identity	Medication(s) used	Medication(s) doses	Duration of treatment	Serum hormone concentrations	Adverse effects
*Feminizing gender-affirming hormonal therapy*							
Wassersug R, Gray RE, Barbara A, Trosztmer C, Raj R, Sinding C. Experiences of Transwomen with Hormone Therapy. Sexualities. 2007 Feb 1;10(1):101–22. [17]	Investigate experiences of 12 transwomen on hormone treatment qualitatively	12 transwomen	Antiandrogen therapy (spironolactone or cyproterone acetate) and/or estrogens (conjugatedestrogens and/or ethinyl estradiol and/or estriol)	Not reported	A minimum of 6 months’ experience and a maximum of 4 years’ experience with hormone treatment	Not reported	Changes in libido, metabolism, and mood reported by a few participants
Fung R, Hellstern-Layefsky M, Lega I. Is a lower dose of cyproterone acetate as effective at testosterone suppression in transgender women as higher doses? Int J Transgenderism. 2017 Apr 3;18(2):123–8. [18]	Determine whether CPA at 25 mg daily would suppress total testosterone as effectively as 50 mg daily in transgender women.	68 transgender women	Cyproterone acetate	Group 1, CPA 25 mg (*N* =31); group 2, CPA 50 mg or higher (*N* = 19); group 3, CPA dose lowered from 50 mg to 25 mg (*N* = 15); group 4, CPA dose increased from 25 mg to 50 mg (*N* = 3)	April 1, 2009 and June 30, 2015	Mean total testosterone on treatment was 0.9 nmol/L (95% CI 0.7 to 1.1) in group 1 and 1.2 nmol/L (95% CI 0.9–1.5) in group 2 and were not significantly different (*p* = 0.087). In group 3, there was no significant difference between total testosterone levels before and after decreasing the dose of CPA from 50 mg to 25 mg, *p* = 0.86. Group 4 was excluded from analysis.	Not reported
Cohen H, Forget H. Auditory cerebral lateralization following cross-gender hormone therapy. Cortex J Devoted Study Nerv Syst Behav. 1995 Sep;31(3):565–73. [19]	Investigate relations between hormone therapy and auditory cerebral specialization for speech and non-speech stimuli	13 genetic male transsexuals	Various medications used which were different for each patient, including: Estinyl, Provera, Tace, Delalutin, Delostrogen, Cebestrole, Pre marine,	Various doses different for each patient and for each medication: please see Table 1 of the referenced paper.	1.3 to 23.8 years	Not reported	Not reported
Lam GY, Goodwin J, Wilcox P, Quon BS. Worsening pulmonary outcomes during sex reassignment therapy in a transgender female with cystic fibrosis (CF) and asthma/allergic bronchopulmonary aspergillosis: a case report. BMC Pulm Med. 2020 Aug 31;20(1):234. [21]	Describe a case of a patient with CF and asthma/allergic bronchopulmonary aspergillosis (ABPA) undergoing sex reassignment therapy (male-to-female) and the negative impact it had on her lung function and frequency of pulmonary exacerbations in the context of increasing doses of exogenous estrogen	1 transgender female	Estrogen, cyproterone	Estrogen 5 mg daily, cyproterone 25 mg daily	1 year	300 pmol/L	Relatively stable patient with CF lung disease complicated by both asthma and allergic bronchopulmonary aspergillosis, who then experienced a rapid decline in lung function and increased frequency of pulmonary exacerbations temporally corresponding to rising serum levels of estradiol as part of ongoing sex reassignment therapy
Prior JC, Vigna YM, Watson D. Spironolactone with physiological female steroids for presurgical therapy of male-to-female transsexualism. Arch Sex Behav. 1989 Feb 1;18(1):49–57. [22]	Describe clinical and hormonal response to 12-month therapy with the antiandrogen spironolactone, in conjunction with near-physiologic doses of female gonadal steroid	50 transsexual males	Spironolactone, estrogen, medroxyprogesterone	Spironolactone: initially 100–200 mg/day and gradually increased; estrogen: 0.625 mg/day, increasing to 2.5 mg twice/day for 3 out of 4 weeks; medroxyprogesterone: 10 mg/day, during weeks 3 and 4 of a 4-week cycle	1 year	Testosterone, dehydroepiandrosterone, prolactin, triglyceride, leutinizing hormone concentrations provided in Table 3 of cited article	Not reported
Hallarn J, Bauer GR, Potter E, Wilcox H, Newfeld J, Krakowsky Y, et al. Gynecological concerns and vaginal practices and exposures among transfeminine individuals who have undergone vaginoplasty. J Sex Med. 2023 Nov 1;20(11):1344–52. [23]	Describe self-reported gynecological concerns and vaginal care practices among transfeminine persons who have undergone vaginoplasty	60 transfeminine participants	Estrogen, progestin	Not reported	1 year	Not reported	Not reported
Armstrong I, Lacombe-Duncan A, Shokoohi M, Persad Y, Tseng A, Fung R, et al. Feminizing hormone therapy in a Canadian cohort of transgender women with and without HIV. Antivir Ther. 2023 Jun 1;28(3):13,596,535,231,182,505. [25]	Characterize patterns of feminizing hormone therapy and antiretroviral therapy among trans women with HIV and to compare serum hormone levels to trans women without HIV	1495 trans women	Oral estradiol, intramuscular estradiol valerate, transdermal estradiol patch, transdermal estradiol gel, spironolactone, cyproterone, bicalutamide, dutasteride, finasteride, leuprolide	Not reported	1 year	Overall median serum estradiol in pmol/L (IQR): 217 (119.5, 401.0); overall serum testosterone in nmol/L (IQR): 0.70 (0.40, 5.0)	Not reported
Davies SM, Johnston JR. Exploring the validity of the Transsexual Voice Questionnaire for male-to-female transsexuals. Can J Speech-Lang Pathol Audiol. 2015;39(1):40–51.[26]	Investigate the content validity of a newly developed tool, the Transsexual Voice Questionnaire, Male to Female, TVQMtF	5 transsexual women	Not specified, “feminizing hormones”	Not reported	5 months to 7 years	Not reported	Not reported
Obiezu CV, Giltay EJ, Magklara A, Scorilas A, Gooren L, Yu H, et al. Dramatic suppression of plasma and urinary prostate specific antigen and human glandular kallikrein by antiandrogens in male-to-female transsexuals. J Urol. 2000 Mar;163(3):802–5.[38]	Determine whether PSA and hK2 change significantly in plasma and urine after antiandrogen treatment in male-to-female transsexuals	31 transsexual males	Ethinyl estradiol, cyproterone acetate	100 µg. oral ethinyl estradiol daily, 100 mg. cyproterone acetate daily	1 year	Levels of PSA and hK2 provided in Tables 1 and 2 of cited article	Not reported
Fung R, Hellstern-Layefsky M, Tastenhoye C, Lega I, Steele L. Differential Effects of Cyproterone Acetate vs. Spironolactone on Serum High-Density Lipoprotein and Prolactin Concentrations in the Hormonal Treatment of Transgender Women. J Sex Med. 2016 Nov 1;13(11):1765–72. [39]	Compare the effects of spironolactone and CPA on HDL and prolactin serum concentrations in transgender women	113 transgender women	Spironolactone or cyproterone acetate in combination with estrogen	Mean dose of spironolactone: 181 mg (SD = 75.0, range = 25–400 mg); mean dose of CPA: 31 mg (SD 10.9, range = 25–50 mg)	1 year	At 12 months, HDL increased by 0.10 mmol/L (SD = 0.24) in the spironolactone group but decreased by 0.07 mmol/L (SD = 0.21) in the CPA group (*P* = 0.002). The change in prolactin was + 3.10 µg/L (SD = 5.70) in the spironolactone group and + 11.8 µg/L (SD = 8.63) in the CPA group (*P* < 0.001).	Not reported
*Masculinizing gender-affirming hormonal therapy*							
Miller N, Bédard YC, Cooter NB, Shaul DL. Histological changes in the genital tract in transsexual women following androgen therapy. Histopathology. 1986 Jul;10(7):661–9. [16]	Examine histological changes in the genital tract in transsexual women following androgen therapy	32 transsexual women	Delatestryl, depo-testosterone	Delatestryl: 200 mg monthly to 400 mg every week intramuscularly (IM), depo-testosterone: 200-400 mg IM every 4 weeks	1 year	Not reported	Not reported
Fung R, Greenaway MK, McEvenue G. Gynecomastia in a Transgender Boy: A Case Report. AACE Clin Case Rep. 2021 Nov 1;7(6):350–2.[28]	Describe the case of a 17-year-old transgender boy who experienced breast development while on testosterone, having been suppressed with a gonadotropin-releasing hormone (GnRH) agonist prior to testosterone therapy	1 transgender boy	Gonadotropin-releasing hormone (GnRH) agonist, testosterone cypionate	Testosterone cypionate: 100 mg IM every 2 weeks	6 years	Total testosterone and estradiol levels in relation to the patient’s testosterone level and onset of gynecomastia, displayed in Table 1 of cited paper	Gynecomastia
Obiezu CV, Giltay EJ, Magklara A, Scorilas A, Gooren LJG, Yu H, et al. Serum and Urinary Prostate-specific Antigen and Urinary Human Glandular Kallikrein Concentrations Are Significantly Increased after Testosterone Administration in Female-to-Male Transsexuals. Clin Chem. 2000 Jun 1;46(6):859–62.[40]	Present serum and urinary PSA and hK2 before, and 4 and 12 months post testosterone treatment by immunofluorometric methods in 32 female-to-male transsexuals	32 female-to-male transsexuals	Testosterone esters	250 mg of testosterone esters (SustamonR; Organon Oss) every 2 weeks	1 year	Mean serum PSA increased from 1.1 ng/L to 11.1 ng/L and then to 22 ng/L by 4 and 12 months post treatment, respectively; serum hK2, remained undetectable at the three time points	Not reported
Todd CM, Yu A, Lay C, Lagman-Bartolome AM. Effect of testosterone therapy on migraine frequency and disability in two transgender patients: a case report. BMJ Case Rep CP. 2023 Jan 1;16(1):e251895.[41]	Report the potential effect of testosterone on migraine among transgender patients	2 transmasculine patients	Testosterone or testosterone enanthate	Testosterone: 5 mg/24 hours patches; testosterone enanthate: 50 mg weekly	1–4 years	Not reported	Migraines
Wilson DM, Kiang TKL, Ensom MHH. Pharmacokinetics, safety, and patient acceptability of subcutaneous versus intramuscular testosterone injection for gender-affirming therapy: A pilot study. Am J Health-Syst Pharm AJHP Off J Am Soc Health-Syst Pharm. 2018 Mar 15;75(6):351–8.[42]	Compare testosterone exposure and tolerability with subcutaneous versus IM administration	14 transgender males	Testosterone estosterone cypionate or enanthate	Testosterone cypionate 100 mg/mL (Depo-Testosterone, Pfizer Canada Inc., Kirkland, Canada) or testosterone enanthate 200 mg/mL (Delatestryl, Valeant Canada LP, Laval, Canada)	11 weeks	Mean ± S.D. Normalized through total serum testosterone concentration (nmol/L/mg) at week 11: 0.29 ± 0.09	Subcutaneous route was more tolerable, with lower self-reported scores for pre-injection anxiety, pain during injection, and post-injection pain
Taillefer V, Kelley J, Marsolais S, Chiniara L, Chadi N. Expected vs. perceived effects of gender-affirming hormone therapy among transmasculine adolescents. J Pediatr Endocrinol Metab. 2023 Nov 1;36(11):1072–8.[43]	Explore expected vs. perceived effects of gender-affirming hormone therapy	76 transmasculine participants	Testosterone cypionate or enanthate	Testosterone IM or subcutaneous form with a starting dose of 25–50 mg of testosterone cypionate or enanthate administered every 2 weeks, with stepwise increases at 6-month intervals up to maximal doses of 50–100 mg weekly or 100–200 mg every 2 weeks	24 months	Mean (SD) serum testosterone levels at 24 months: 22.30 (8.74)	Not reported
Kogachi K, Konstas A, Karanjia R, Sadun AA. Endovascular Stenting in a Transgender Patient With Idiopathic Intracranial Hypertension. J Neuro-Ophthalmol Off J North Am Neuro-Ophthalmol Soc. 2019 Jun;39(2):247–8.[44]	Present a case of a transgender patient with idiopathic intracranial hypertension (IIH)	1 female-to-male transgender patient	Testosterone cypionate	100 mg injected IM every week	50 months	Not reported	Unable to conclusively determine whether the etiology of the stenosis was extrinsic secondary to elevated ICP or intrinsic due to the anatomy of the venous sinus
Oberc A, Armstrong K, Ko HM, Grant A, Mullen JBM, Williams P. Case report of a breast granular cell tumor in a young transgender man. Int J Surg Case Rep. 2022 Apr 1;93:106978.(45)	Report an unusual case of a breast granular cell tumor in a young transgender man	1 transgender male	Testosterone therapy	Not reported	1 year	Not reported	Not reported
Nayman T, Hébert M, Ospina LH. Idiopathic intracranial hypertension in a pediatric transgender patient. Am J Ophthalmol Case Rep. 2021 Dec 1;24:101208.[46]	Describe the first case of IIH in a transgender patient	1 female-to-male transgender patient	Testosterone	50 mg/week	10 months	Not reported	Not reported
*Puberty blockers*							
Pullen Sansfaçon A, Temple-Newhook J, Suerich-Gulick F, Feder S, Lawson ML, Ducharme J, et al. The experiences of gender diverse and trans children and youth considering and initiating medical interventions in Canadian gender-affirming speciality clinics. Int J Transgenderism. 2019 Oct 2;20(4):371–87.[24]	Develop a deeper understanding of experiences of trans youth seeking and receiving gender-affirming care at Canadian specialty clinics, including their goals in accessing care, feelings about care, and medical interventions they have undergone, and whether they have any regrets about these interventions	35 trans and gender diverse young people	Puberty blockers	Not reported	Not reported	Not reported	Hot flashes, mood swings, headaches
Navabi B, Tang K, Khatchadourian K, Lawson ML. Pubertal Suppression, Bone Mass, and Body Composition in Youth With Gender Dysphoria. Pediatrics. 2021 Oct 1;148(4):e2020039339. [29]	Examine the effects of gonadotropin-releasing hormone agonists (GnRHa) on bone mass and body composition among youth with gender dysphoria	172 youth with gender dysphoria	Leuprolide acetate	3 doses of 7.5 mg intramuscularly every 4 weeks, followed by 11.25 mg intramuscularly every 12 weeks	Not reported	Not reported	Decreased bone mineral density without evidence of fractures or changes in BMI z score, vitamin D insufficiency or deficiency
Heard J, Morris A, Kirouac N, Ducharme J, Trepel S, Wicklow B. Gender dysphoria assessment and action for youth: Review of health care services and experiences of trans youth in Manitoba. Paediatr Child Health. 2018 May 11;23(3):179–84. [20]	Describe the pediatric transgender population accessing health care through the Manitoba Gender Dysphoria Assessment and Action for Youth (GDAAY) program, and report youth’s experiences accessing health care in Manitoba	122 natal females and 52 natal males	Leuprolide acetate, medroxyprogesterone acetate	Not reported	Not reported	Not reported	Not reported
Khatchadourian K, Amed S, Metzger DL. Clinical management of youth with gender dysphoria in Vancouver. J Pediatr. 2014 Apr;164(4):906–11.[30]	Describe patient characteristics at presentation, treatment, and response to treatment in youth with gender dysphoria	84 youth with gender dysphoria	Gonadotropin-releasing hormone analog	Not reported	Not reported	Not reported	1 FtM patient developed sterile abscesses and was switched from leuprolide acetate to triptorelin, which was tolerated well; 1 FtM patient developed leg pains and headaches, which eventually resolved without treatment; 1 patient gained 19 kg within 9 months of initiating GnRHa, although the patient’s body mass index was > 85 percentile before initiation of GnRHa
Zucker KJ, Bradley SJ, Owen-Anderson A, Singh D, Blanchard R, Bain J. Puberty-Blocking Hormonal Therapy for Adolescents with Gender Identity Disorder: A Descriptive Clinical Study. J Gay Lesbian Ment Health. 2010 Dec 29;15(1):58–82.[31]	Examine demographic behavioral problems and psychosexual measures to see if any of them correlate with the clinical decision to recommend, or not recommend, puberty-blocking hormonal therapy	109 adolescents with gender identity disorder	Puberty blockers	Not reported	Not reported	Not reported	Not reported
Bauer GR, Lawson ML, Metzger DL. Do Clinical Data from Transgender Adolescents Support the Phenomenon of “Rapid Onset Gender Dysphoria”? J Pediatr. 2022 Apr 1;243:224–227.e2.[32]	Investigate the potential of a new etiologic phenomenon of rapid onset gender dysphoria during adolescence	173 youth referred for hormonal suppression	Hormonal suppression	Not reported	Not reported	Not reported	Not reported
Waldner RC, Doulla M, Atallah J, Rathwell S, Grimbly C. Leuprolide Acetate and QTc Interval in Gender-Diverse Youth. Transgender Health. 2023 Feb;8(1):84–8.	Determine the proportion of gender-diverse youth with QTc prolongation on leuprolide acetate therapy	33 gender diverse youth	Leuprolide acetate	7.5 mg intramuscular (IM) injections once per month	Not reported	Not reported	Not reported
Silva C, Fung A, Irvine MA, Ziabakhsh S, Hursh BE. Usability of Virtual Visits for the Routine Clinical Care of Trans Youth during the COVID-19 Pandemic: Youth and Caregiver Perspectives. Int J Environ Res Public Health. 2021 Jan;18(21):11,321. [48)	Evaluate families’ perspectives on usability of virtual visits for routine gender care for trans youth during the COVID-19 pandemic	28 trans youth	Puberty blockers	Not reported	Not reported	Not reported	Not reported
*Lactation and Fertility*							
Jones CA, Reiter L, Greenblatt E. Fertility preservation in transgender patients. Int J Transgenderism. 2016 Apr 2;17(2):76–82. [27]	Identifying characteristics of the transgender patient population and the barriers to successful gamete banking	11 male-to-female (MtF) transgender patients and 3 female-to-male (FtM) transgender patients	Hormone therapy	Not reported	Length of time on hormone therapy was recorded in 6 of the 11 MtF patients’ charts; of these 6 patients, the mean length of time on hormones prior to visiting the clinic was 0.33 months compared with 35.2 months for the 3 FtM patients	Not reported	Not reported
Wamboldt R, Shuster S, Sidhu BS. Lactation Induction in a Transgender Woman Wanting to Breastfeed: Case Report. J Clin Endocrinol Metab. 2021 Apr 23;106(5):e2047–52.[36]	Review potential methods of lactation induction in trans women wishing to breastfeed	1 transgender woman	Progesterone and galactogogue domperidone	Daily dose of progesterone was increased from 100 mg to 200 mg daily. The galactogogue domperidone was started at 10 mg 3 times daily and titrated up to effect	9 months	Not reported	Not reported
*Various or unreported combinations of hormone therapy*							
Bauer GR, Pacaud D, Couch R, Metzger DL, Gale L, Gotovac S, et al. Transgender Youth Referred to Clinics for Gender-Affirming Medical Care in Canada. Pediatrics. 2021 Nov;148(5):e2020047266.[15]	Describe characteristics of trans youth in Canada at first referral visit	137 were transmasculine (assigned female) and 37 transfeminine (assigned male) youth	Depot leuprolide acetate, testosterone, estrogen, spironolactone, medroxyprogesterone acetate	Not reported	Not applicable	Not reported	Not reported
Lacombe-Duncan A, Newman PA, Bauer GR, Logie CH, Persad Y, Shokoohi M, et al. Gender-affirming healthcare experiences and medical transition among transgender women living with HIV: a mixed-methods study. Sex Health. 2019 Jul 9;16(4):367–76.[33]	Describe barriers and facilitators to access to medical transition among transgender women living with HIV	48 trans women	Unspecified, “hormone therapy”	Not reported	Not reported	Not reported	Not reported
Clark BA, Veale JF, Townsend M, Frohard-Dourlent H, Saewyc E. Non-binary youth: Access to gender-affirming primary health care. Int J Transgenderism. 2018 Apr 3;19(2):158–69.[34]	Document differences in access to gender-affirming health care between binary and non-binary identified trans youth and explore ways of meeting the health needs of non-binary youth within primary care settings	344 non-binary youth	Unspecified, “hormone therapy”	Not reported	Not reported	Not reported	Not reported
Hardy TLD, Rieger JM, Wells K, Boliek CA. Acoustic Predictors of Gender Attribution, Masculinity-Femininity, and Vocal Naturalness Ratings Amongst Transgender and Cisgender Speakers. J Voice Off J Voice Found. 2020 Mar;34(2):300.e11-300.e26.[35]	Identify the most salient set of acoustic predictors of (1) gender attribution; (2) perceived masculinity–femininity; and (3) perceived vocal naturalness among a group of transgender and cisgender speakers	22 transgender women	Unspecified “hormone replacement therapy”	Not reported	Not reported	Not reported	Not reported
El-Hadi H, Stone J, Temple-Oberle C, Harrop AR. Gender-Affirming Surgery for Transgender Individuals: Perceived Satisfaction and Barriers to Care. Plast Surg. 2018 Nov;26(4):263–8.[47]	Examine the perceived satisfaction and barriers to care for transgender patients after they decide to undergo gender-affirming surgery	12 female to male and 20 as male to female	“Various combinations of hormonal therapy”	Not reported	Not reported	Not reported	Not reported

### Outcomes after gender-affirming hormone treatment organized by treatment received

#### Testosterone treatment

In total, nine studies focused on testosterone treatment [16, 28, 40–46]. Among these studies, four measured physiologic parameters, two measured physiologic parameters and provider-reported outcomes, one measured patient-reported outcomes, and two measured physiologic parameters and patient-reported outcomes.

In terms of physiologic parameters, in one study, Obiezu et al. [40] treated patients with testosterone, 250 mg intramuscularly, every 2 weeks and collected serum and urine samples to measure testosterone, PSA (prostate-specific antigen), and hK2 (human glandular kallikrein) [40]. They found that testosterone administration significantly increased PSA in serum and urine and hK2 in urine by the fourth month of treatment [40]. In another study, Miller et al. [16] examined the effects of testosterone therapy on histological examination following hysterectomy [16]. They identified marked atrophy of the cervical epithelium, which could mimic dysplasia, and variable degrees of atrophy of the endometrium [16]. They also determined that ovaries showed occasional corpora lutea, indicating that ovulation may occur despite testosterone therapy [16]. In another study, Kogachi et al. [44] described a case of a patient on testosterone cypionate, 100 mg intramuscularly per week, who developed transient obscuration of vision, headache, dizziness, and “ear popping” [44]. The patient had papilledema and elevated cerebrospinal fluid opening pressure. The patient was diagnosed with idiopathic intracranial hypertension and was managed with endovascular stenting, without reduction to testosterone therapy. The authors discussed the role of hormones in the associations between transverse sinus stenosis and increased intracranial pressure [44]. In another study, Nayman et al. described a case of a patient on testosterone, 70 mg per week, who developed transient obscuration of vision and frontal headache. The patient, who also had papilledema and elevated cerebrospinal fluid opening pressure, was diagnosed with idiopathic intracranial hypertension and was managed with acetazolamide and reduction of testosterone therapy [46]. In another study, Oberc et al. [45] described a case of a patient who was on testosterone and developed a painless palpable mass in the right breast, which was found to be a granular cell tumor (GCT) [45]. The authors commented that such tumors are rare and poorly understood, particularly in transgender individuals, in whom they have not been previously documented and that they may mimic other benign or malignant lesions [45]. In another study, Fung et al. [28]. described a case of a patient on testosterone who developed gynecomastia [28],. The authors underlined that clinicians should be aware that transgender boys undergoing testosterone therapy may develop gynecomastia and that adjustment of testosterone therapy could lead to improvements [28].

In terms of patient-reported outcomes, in one study, Taillefer et al. [43] demonstrated that the most desired outcomes of testosterone therapy in transmasculine individuals was increased body/facial hair (69% of study sample) and voice deepening/Adam’s apple growth (52% of study sample) [43]. These effects were reached in 80% of reports at 12 months, continuing to increasing steadily across the 24-month follow-up period [43]. In another study, Wilson et al. [42] investigated self-reported pre-injection anxiety, peri-injection pain, and post-injection pain for intramuscular (IM) testosterone versus subcutaneous testosterone [42]. The study found that the subcutaneous route was preferred by participants and that it resulted in similar testosterone concentration to that of the intramuscular route [42]. In another study, Todd et al. [41] described two cases of testosterone therapy that had a positive impact on headache frequency and intensity [41].

### Estrogen and antiandrogen treatments

In total, ten studies focused on estrogen and/or antiandrogen treatment [17–19, 21–23, 25, 26, 38, 39]. Among these studies, six measured physiologic parameters, three measured patient-reported outcomes, and one measured physiologic parameters and provider-reported outcomes.

In terms of physiologic parameters, in one study, Fung et al. [18] compared different doses of cyproterone acetate combined with estrogen and found that lower doses (25 mg) were as effective as higher doses (≥ 50 mg) in suppressing testosterone concentrations to achieve the normal female range when used in combination with recommended estrogen therapy [18]. In another study, Prior et al. [22] examined the effects of spironolactone combined with low-dose estrogen. They compared a group of patients who were treatment-naïve with a group of patients who had previously received high-dose estrogen monotherapy. They noted a significant decrease in testosterone concentrations of both groups relative to their baselines. They also observed decreased male pattern hair loss and breast development [22]. In another study, Armstrong et al. [25] explored the effects of antiretroviral therapy on feminizing hormone therapy. Comparing patients on antiretroviral therapy with those not on antiretroviral therapy, they noted no significant differences in serum estradiol and testosterone concentrations between the two [25]. In another study, Fung et al. [39] studied the effects of spironolactone versus cyproterone acetate (CPA) on serum high-density lipoprotein (HDL). They found that spironolactone use increases HDL concentration, whereas CPA decreases HDL concentrations [39]. In another study, Obiezu et al. [38] examined the effects of cyproterone acetate, alone or in combination with estrogen, on plasma and urinary PSA and hK2 concentration. They found that CPA, both alone and in combination with estrogen, suppresses > 90% of plasma and urinary PSA and hK2 concentration after 4 or 12 months of therapy [38]. In another study, Cohen et al. [19] investigated associations between hormone therapy and auditory cerebral specialization for speech and non-speech stimuli. They found that cisgender women and transgender women exhibited similar patterns in verbal and nonverbal tasks, with a right ear advantage for verbal tasks and without a left ear advantage for nonverbal tasks, which was also exhibited in cisgender men. The authors suggest the possible role of hormones in right hemispheric cognitive processing [19]. In another study, Lam et al. [21] describe a case of a patient with asthma and cystic fibrosis who experienced a decrease in lung function with an increase in estrogen [21].

In terms of patient-reported outcomes, one study by Davies and Johnston [26] was involved in developing a PROM, the Trans Voice Questionnaire [26]. In another study, Wassersug et al. investigated the effects of antiandrogen therapy and estrogen therapy, noting an overall improvement in mental health outcomes [17]. The study found that antiandrogen and/or estrogen therapy was seen as the critical step in committing to and consolidating gender transition [17].

### Puberty blockers (with or without testosterone or estrogen)

In total, eight studies focused on puberty blockers [20, 24, 29–32, 37, 48]. Among these studies, two measured physiologic parameters, one measured physiologic parameters, patient-reported outcomes, and provider-reported outcomes, four measured patient-reported outcomes, and one measured on patient-reported outcomes and provider-reported outcomes.

In terms of physiologic parameters, in one study, Waldner et al. [37] examined the effects of gonadotropin-releasing hormone agonist (GnRHa) (specifically, leuprolide acetate) on the QT interval. They noted no significant increase of the QT interval [37]. In another study, Navabi et al. [29] studied the effects of GnRHa on bone mass and body composition. They observed a reduction in bone mineral density (without evidence of fractures) and a redistribution of body fat in gynoid and android pattern, respectively [29]. In another study, Khatchadourian et al. [30] scrutinized the effects of GnRHa followed by hormone therapy. The use of both was well tolerated overall; however, of those using GnRHa, one developed an abscess, another a headache and leg pain, and another significant weight gain. Conversely, among those using hormones, seven developed severe acne, three mild dyslipidaemia, one androgenetic alopecia, and one mood swings [30].

In terms of patient-reported outcomes, in one study, Zucker et al. [31] explored the decision-making process around recommendations for puberty blockers, recognizing the role of demographic, behavioral, and psychosexual measures on these recommendations. The studied noted that having more cross gender-behavior, more self-reported gender dysphoria, and lower behavior problem scores, among some other measures, is associated with recommendations for puberty blockers [31]. In another study, Bauer et al. [32] investigated the idea of rapid onset gender dysphoria, which suggests that some adolescents suddenly experience gender dysphoria due to societal influences. The study found no significant associations between recency of gender knowledge and mental health issues. In fact, the study found that recent gender knowledge was associated with lower anxiety and lower marijuana use [32]. In another study, Heard et al. [20] examined the experiences of children and adolescents accessing gender-affirming care, including puberty blockers and hormones, in Manitoba. They reported that 70% of individuals had to provide some education to a healthcare provider regarding their needs as a transgender patient. In addition, they reported that 65% of individuals had been told that their healthcare provider did not possess adequate knowledge about transgender-related care to provide it. In another study, Silva et al. [48] studied the experiences of children, adolescents, and their caregivers via virtual visits during the pandemic, including those receiving puberty blockers and hormones, and noted that 100% of participants felt that virtual visits met their healthcare needs and deemed virtual visits to be safe or safer than in-person visits. The study reported that 94% of participants wanted to continue using virtual visits after the pandemic [48]. In another study, Pullen et al. [24] explored the experiences of children and adolescents accessing gender-affirming care. They study observed improvements in wellbeing after starting medical interventions, including puberty blockers and hormones. The study noted that no participants experienced regret regarding their medical interventions, although some did experience side effects [24].

### Studies reporting various hormone treatments

In total, five studies did not specify the hormonal treatment or used various combinations of hormonal treatments that did not clearly focus on one of the above areas [15, 33–35, 47]. Among these studies, two measured physiologic parameters and patient-reported outcomes, one measured provider-reported outcomes and patient-reported outcomes, and two measured patient-reported outcomes.

In terms of physiologic parameters, in one study, Bauer et al. [15] assessed characteristics of trans children and adolescents at their first referral visit for pubertal suppression or hormone therapy. They identified high rates of anxiety and past-year self-harm [15]. In another study, Hardy et al. [35] aimed to identify acoustic predictors of gender attribution, masculinity-femininity, and vocal naturalness in individuals who had received hormone therapy gender-affirming surgery (GAS), and in some, additional forms of gender-affirming care. They found that fundamental frequency (f_o_) was a predictor of gender attribution with higher fundamental frequency associated with a higher chance of being perceived as female. They also determined that mean fundamental frequency, formant frequency, and sound pressure level predicted masculinity-femininity, while mean fundamental frequency, formant frequency, and speech rate predicted vocal naturalness [35].

In terms of patient-reported outcomes, a study by Lacombe-Duncan et al. [33] aimed to identify barriers and facilitators to accessing medical transition among transgender women living with HIV (human immunodeficiency virus). They found that over half of participants were undergoing or had completed medial transition, primarily with hormone therapy. They identified barriers, including stigma and other social determinants of health [33]. In another study, Clark et al. [34] aimed to identify barriers to accessing medical transition for non-binary youth relative to binary youth. They found that non-binary youth were significantly less likely to access necessary healthcare compared to binary youth [34]. They also found that non-binary youth were significantly less likely to access hormone therapy, but were more likely to report experiencing barriers to accessing hormone therapy compared to binary youth [34]. In another study, El-Hadi et al. [47] aimed to assess perceived satisfaction and barriers to gender-affirming surgery. They found that gender-affirming surgery, often combined with hormonal therapy, was important for the quality of life of 91% of participants. They also found that 100% of participants were happy with their decision to undergo GAS [47]. The same study identified the following barriers to gender-affirming surgery: financial (73%), finding a physician (65%), and access to information (63%) [47].

### Fertility and lactation

In terms of fertility preservation, one study by Jones et al. [27] assessed patients initiating cross-sex hormones, measuring physiologic parameters. They found that female-to-male transgender patients were referred for fertility preservation at a later age and after a longer duration of hormone therapy compared to male-to-female transgender patients [27]. In total, none of the female-to-male transgender patients underwent any cryopreservation techniques; however, nine of the eleven male-to-female transgender patients successfully cryopreserved sperm [27]. The authors highlighted the need to increase awareness of age effects on fertility among transgender patients and to increase access to fertility preservation technologies for transgender people early in their transition [27].

In terms of lactation induction, one study by Wamboldt et al. [36] measured provider-reported outcomes [36]. This study described a case of successful lactation induction in a transgender woman who received progesterone at 200 mg daily [36]. The galactagogue, domperidone, was started at 10 mg three times daily and titrated up to effect [36]. The patient was encouraged to use an electric pump and, at 1 month, noticed significant increase in breast size and fullness [36]. The patient was able to produce 3 to 5 ounces of milk per day with manual expression [36].

### Quality assessment

The CASP and the JBI checklists were applied to assess the quality of the included articles.

The CASP checklist was applied in 27 studies. The studies all (100%) addressed a clearly focused issue and recruited cohorts in an acceptable way. The majority of studies (96%) accurately measured the exposures and outcomes to minimize bias. The exposures and outcomes were accurately measured in most studies (96%), minimizing bias. However, the identification of all important confounding factors occurred in only half of the studies (48%), while the consideration of important confounding factors in design and/or analysis also occurred in only half of the studies (52%). The follow-up of participants was considered complete enough and long enough in 63% of studies. The results were considered believable, applicable to the local population, and fitting with other available evidence in 96%, 100%, and 100% of studies, respectively.

The JBI checklist was applied to seven case reports. The case reports all (100%) clearly described patient demographics, history presented as a timeline, current clinical condition, diagnostic tests, interventions or treatment procedures, and post-intervention clinical condition and provided takeaway lessons. The majority of case reports (71%) identified and described adverse or unanticipated events.

The above information is presented in the Supplementary Tables.

## Discussion

This study presents the outcomes of gender-affirming hormone treatments in Canada. Overall, the reporting of outcome measurements was inconsistent across studies, which made it difficult to compare across them. However, it was demonstrated that, on the whole, patients had positive impacts from gender-affirming hormone treatments.

It is important that outcome measurement for gender-affirming care comprehensively measure patient perspectives [1]. In this study, PROMs were infrequently used, which can create issues as regards understanding patient perspectives as part of care. When PROMs were utilized, various instruments were used with a lack of standardization. In order to minimize research waste, it is important to have a consistent outcome measurement strategy for gender-affirming hormone treatments. This strategy should be balanced between physiologic parameters, provider-reported outcomes, and patient-reported outcomes. There are a number of potentially useful PROMs, including the Gender Congruence and Life Satisfaction Scale [68] and the iTransQoL [69], which was developed specifically for gender-affirming hormone therapy. For assistance with PROMs implementation, clinicians and researchers may wish to review the guidance offered by the Practical Guide to Implementing PROMs in Gender-Affirming Care (PG-PROM-GAC) [70, 71]. This is an evidence-based resource that provides information on increasing PROMs uptake in various settings.

In addition to inconsistency in the reporting of outcomes, there was also inconsistency in the reporting of race and gender. In fact, no studies used the two-step method (asking about gender and then sex assigned at birth) when collecting gender identity information [72]. This is a significant limitation as it is important to ensure that the gender of patients is captured accurately and in their own words rather than imposed by healthcare providers, as was observed in several studies. There was also limited data from some provinces and cities in Canada as most studies were conducted in the province of Ontario and the city of Toronto. This points to a potential need to increase access to and research on gender-affirming hormone treatments in Canada, particularly outside of Toronto, Vancouver, and Montreal.

Strengths of this study include a comprehensive search with minimal limitations and broad inclusion to capture as many articles as possible relating to gender-affirming hormone treatment in Canada. In addition, the involvement of patient partners in this study aids to ensuring its relevance and applicability to the enhancement of patient care. Limitations of this study include the fact that despite the comprehensive search, some articles may have been missed. Furthermore, due to inconsistency in reporting between studies (concerning concepts such as gender, race/ethnicity, and outcomes), it was difficult to perform a cohesive synthesis and comparison between studies. Future research should focus on developing a more consistent outcome measurement and evaluation strategy for gender-affirming hormone treatment in Canada.

## Conclusion

The current systematic review presents the outcomes after gender-affirming hormone treatments in Canada. In general, outcomes were positive for patients. However, there was significant variation in the reporting of race, gender, and outcomes, which complicated the undertaking of comparisons between studies. This underscores the need to improve standardization of outcome measurement for gender-affirming hormone treatments in order to improve the robustness of the evidence base, thereby contributing to higher quality research and evidence-informed clinical practice. There is also a need to consider patient-reported outcome measures, which were often overlooked, despite their being a crucial component of providing high-quality gender-affirming care.

## Electronic supplementary material

Below is the link to the electronic supplementary material.

Supplementary Material 1

## Abbreviations

PROM: Patient-reported outcome measure
TGD: Transgender and gender diverse
